# Supplementary material for: A New Paclitaxel Formulation Based on Secretome Isolated from Mesenchymal Stem Cells Shows a Significant Cytotoxic Effect on Osteosarcoma Cell Lines
Source: Pharmaceutics. 2023 Sep 19;15(9):2340. doi: 10.3390/pharmaceutics15092340 (PMC10537652; doi:10.3390/pharmaceutics15092340)
Supplement: Supplementary file 1 [file pharmaceutics-15-02340-s001.zip › pharmaceutics-2556191-supplementary.pdf]

Supplementary materials

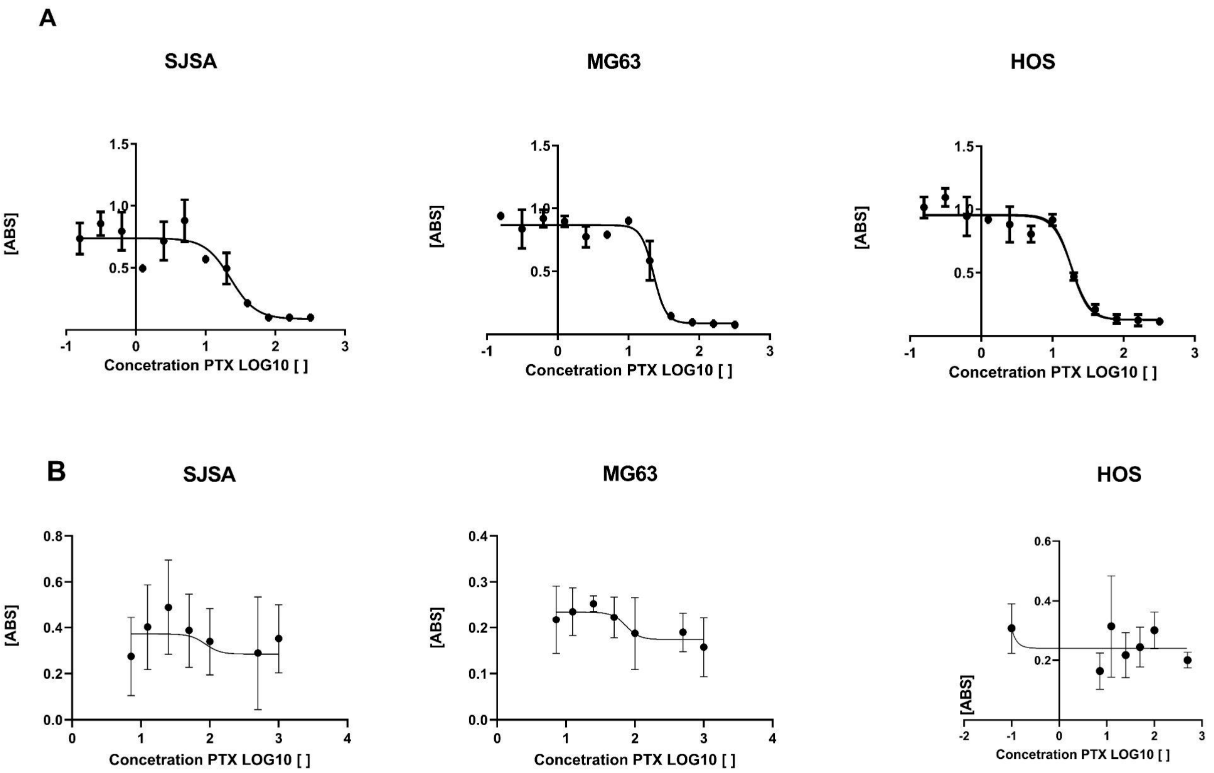

Supplementary Figure S1. IC50 values calculated from the fitted dose–response curves with scalare doses of PTX on SJSA, MG63 and HOS, after 24 h (panels **A**) and after 5 days (panels **B**) from 3 experiments. The values obtained by GraphPad software were shown in table 2 and the error bars in the graphs represent standard deviations.
